# RNA Aptamer Targeting of Adam8 in Cancer Growth and Metastasis

**DOI:** 10.3390/cancers15123254

**Published:** 2023-06-20

**Authors:** Zhiyong Mi, Marissa C. Kuo, Paul C. Kuo

**Affiliations:** 1 Department of Surgery, University of South Florida, Tampa, FL 33620, USA; zhiyong1@usf.edu; 2 Department of Surgery, Vanderbilt University, Nashville, TN 37232, USA; marissa.kuo@vumc.org

**Keywords:** aptamer, Adam8, cancer, metastasis, myCAF

## Abstract

**Simple Summary:**

An RNA aptamer targeting the extracellular sheddase domain of Adam8 was isolated and characterized both in vitro and in vivo. In co-cultures of human mesenchymal stem cells with human MDA-MB-231 breast cancer or Hep G2 cell liver cancer cells, the aptamer blocked extracellular Adam8 activities with associated reversal of the previously established cancer-derived osteopontin-induced myofibroblast cancer-associated fibroblast phenotype (myCAF). Our results suggest that the signal pathways that initiate the development of the myCAF phenotype may be distinct from those required for maintenance, that extracellular Adam8 sheddase activity is required for maintenance of the myCAF phenotype, and that this aptamer may serve as a vehicle for the further investigation of this new pathway.

**Abstract:**

Cancer progression depends on an accumulation of metastasis-supporting physiological changes, which are regulated by cell-signaling molecules. In this regard, a disintegrin and metalloproteinase 8 (Adam8) is a transmembrane glycoprotein that is selectively expressed and induced by a variety of inflammatory stimuli. In this study, we identified Adam8 as a sox2-dependent protein expressed in MDA-MB-231 breast cancer cells when cocultured with mesenchymal-stem-cell-derived myofibroblast-like cancer-associated fibroblasts (myCAF). We have previously found that myCAF-induced cancer stemness is required for the maintenance of the myCAF phenotype, suggesting that the initiation and maintenance of the myCAF phenotype require distinct cell-signaling crosstalk pathways between cancer cells and myCAF. Adam8 was identified as a candidate secreted protein induced by myCAF-mediated cancer stemness. Adam8 has a known sheddase function against which we developed an RNA aptamer, namely, Adam8-Apt1-26nt. The Adam8-Apt1-26nt-mediated blockade of the extracellular soluble Adam8 metalloproteinase domain abolishes the previously initiated myCAF phenotype, or, termed differently, blocks the maintenance of the myCAF phenotype. Consequently, cancer stemness is significantly decreased. Xenograft models show that Adam8-Apt-1-26nt administration is associated with decreased tumor growth and metastasis, while flow cytometric analyses demonstrate a significantly decreased fraction of myCAF after Adam8-Apt-1-26nt treatment. The role of soluble Adam8 in the maintenance of the myCAF phenotype has not been previously characterized. Our study suggests that the signal pathways for the induction or initiation of the myCAF phenotype may be distinct from those involved with the maintenance of the myCAF phenotype.

## 1. Introduction

The growth and metastasis of cancer cells are regulated by reciprocal cross talk between the tumor microenvironment (TME) and cancer stem cells. The TME consists of highly complex and dynamic molecules, blood vessels, and various other cell types, which surround the cancer cell. One key component of the TME is the cancer-associated fibroblast (CAF), which executes multiple functions in order to manipulate cancer development, including facilitating extracellular matrix remodeling, accelerating angiogenesis, promoting the epithelial–mesenchymal transition of cancer stem cells, increasing cancer cells invasion and metastasis, and inducing the ability of tumor cells to evade immunosurveillance and develop resistance to chemotherapies. Molecular drivers that originate from, and are involved in, the TME–cancer stem cell interaction network are ideal targets in either diagnostic or in therapeutic clinical practice.

Adam8 is a transmembrane glycoprotein that is selectively expressed and induced by a variety of inflammatory stimuli [1]. It comprises 824 amino acids with a prototypical N-terminal prodomain; a metalloproteinase-, disintegrin- and cysteine-rich, epidermal-growth factor-like transmembrane domain; and a cytoplasmic tail [2]. Upon autocatalytic prodomain removal, the 90 kDa active form of Adam8 is processed to release a 30 kDa soluble metalloproteinase domain resulting in a 60 kDa remnant on the cell surface [3]. While the expression of Adam8 under normal circumstances is minimal, Adam8 can be upregulated in a variety of pathologic conditions, including asthma, liver injury, and, most notably, cancer [4]. Increased expression of Adam8 has been correlated with enhanced tumor growth and metastasis in breast, pancreatic, liver, colon, and kidney cancers, among others. However, the mechanism by which Adam8 abets cancer potentiation is unknown.

RNA aptamers are small-structured single-stranded RNAs that are established alternatives to antibody-based therapy for the treatment of cancer [5,6,7,8]. RNA aptamers bind to their target proteins with high affinity, are quite stable, and lack immunogenicity. Aptamers are developed by means of an iterative selection method called SELEX (systematic evolution of ligands by exponential enrichment). The shed 30 kDa soluble metalloproteinase domain of Adam8 represents an ideal target for RNA-aptamer-mediated inhibition [9].

We hypothesize that targeting Adam8 in the extracellular space using RNA aptamer technology can inhibit the growth and metastasis of cancer cells. In this study, we sought to utilize the MDA-MB-231 human breast cancer and HepG2 human liver cancer cell lines to characterize the pharmacokinetic and pharmacodynamic properties of an Adam8-directed RNA aptamer (Adam8-Apt-1-26nt) and demonstrate its effect on in vitro and in vivo measures of cancer growth and metastasis.

## 2. Methods

Materials. Human-α-SMA-promoter-driven BFP reporter in human MSC cells: the pCDH-CMV-EF1-Puro lentiviral vector containing human α-SMA promoter (262 bp) with an enhancer (123 bp) was kindly provided by Dr. Shading Bao’s lab (Lerner Research Institute, Cleveland, OH, USA). The mcherry coding region was replaced with the BFP coding region cloned from the plasmid of pCAGGs-BFP (plasmid#127348, Addgene, Watertown, MA, USA) and confirmed by DNA sequencing. The Lentivirus Transduction Enhancer kit (GenTarget Inc., San Diego, CA, USA ) was used to generate human-α-SMA-promoter-driven BFP reporter in human MSC cells [10].

Cell culture: Human mesenchymal stem cells (MSCs) were obtained from the Texas A&M Institute and maintained in Minimal Essential Medium (MEM) media with 20% fetal bovine serum. Human breast cancer cells MDA-MB-231 were obtained from ATCC (Manassas, VA, USA) and maintained in Leibovitz’s L-15 medium (ATCC 30-2008). All cells were cultured in 5% CO_2_ incubator at 37 °C. MDA-MB-231 cells were transfected with Sox2 shRNA lentiviral particles (Santa Cruz Biotechnology, Dallas, TX, USA, sc-38408-v) to constitutively knockdown Sox-2 for use in the co-culture system (confirmed via both real-time PCR and Western blot).

Co-culture: For all co-culture experiments, tumor cells and MSCs in a 1:1 ratio were plated in Boyden Chamber (Corning Inc., Corning, NY, USA) wells with 0.4 μm pores that allow cytokine and growth factor passage but prevent cell movement.

Whole-transcriptome sequencing: The total RNA from MDA-MB-231 cells or MDA-MB-231 (Sox2-KD) cells in co-culture was extracted with RNeasy mini kit (QIAGEN, Germantown, MD, USA) according to the manufacturer’s protocol. The total RNA was sent to NUSeq Core facility of Northwestern University to perform RNA sample integrity assessment, cDNA library preparation, whole-transcriptome sequencing (50 bp; paired-end; 300 M Read, Evanston, IL, USA, and data analysis. Triplicate total RNA samples were extracted from MDA-MB-231 or MDA-MB-231 (Sox2-KD) cells of the following groups:

1. MDA-MB-231 (72 h culture);
2. MDA-MB-231 + MSC (72 h co-culture in the Boyden Chamber);
3. MDA-MB-231(Sox2-KD) + MSC (72 h co-culture in the Boyden Chamber).

Profiling of myCAF-induced cancer-stemness-related genes: We first identified myCAF-dependent gene expression in cancer cells by comparing group 2 to group 1 in terms of increased or newly expressed genes (*p* < 0.05). We then identified the myCAF-induced sox2-dependent genes by determining which genes decreased or were no longer present by comparing group 3 to group 2 (*p* < 0.05). There are 104 genes common to the two comparison groups, thus rendering them myCAF-induced cancer-stemness-related genes.

Profiling of secreted myCAF-induced cancer-stemness-related genes: By searching these 104 genes on the human cancer secretome database (176.58.113.186), we identified 9 genes that encode secreted myCAF-induced cancer-stemness-related proteins. By searching for these 9 genes in a sox2-regulated gene expression database on the genecards.org website, we narrowed the search to 3 genes (Adam8, CA12 and CDH6) representing strong candidates for secreted myCAF-induced cancer-stemness-related genes.

Aptamer selection: We have previously published detailed protocols in which a DuraScribe kit (Biosearch Technologies, Petaluma, CA, USA) and a 40 bp DNA aptamer library (Alpha Diagnostic International, San Antonio, TX, USA) were used to generate an RNA pool [11]. Recombinant human Adam8 protein (Ile-17 to Pro497, Acro Biosystems, Newark, DE, USA) was processed with thermolysin cleavage in vitro to remove its Pro-domain in accordance with the manufacturer’s manual. The his-tag c-terminal-labeled human Adam8 metallo-proteinase domain was applied in the aptamer SELEX selection. A negative selection to remove filter-binding aptamers was performed through Nitrocellulose filter (0.45 μm, Schleicher & Schuell, Keene, NH, USA) incubated with the RNA pool in PBS buffer at 37 °C for 4 h. Under the same conditions, 5 μM protein and 50 μM RNA pools were incubated for 4 h, and the protein/aptamer complex was recovered through the filter flow and using phenol/choloroform extraction/ethanol precipitation. For each round of selection, the protein/aptamer binding affinity was quantified using a competition assay.

Binding affinity assays: Ni-NTA-coated 96-well plates were coated with activated recombinant human Adam8 soluble domain his-tag protein. Cy3-labeled Adam8 aptamers were synthesized via IDT (Integrated DNA Technologies, Coralville, IA, USA). Cy3-Adam8 aptamers were added to the Adam8-Ni-NTA plates at different concentrations in PBS solution at room temperature for 30 min, washed 3 times with PBS, and quantified with Cytation 1 (BioTek, Santa Clara, CA, USA) [12]

Adam8, CA12, and CDH6 genes’ knockdown in MDA-MB-231 cells: Human breast cancer MDA-MB-231 cells were transfected with Adam8/CA12/CDH6 siRNA mixture or their individual shRNA lentiviral particles (Santa Cruz Biotechnology, sc-41406/v, sc-41463/v, sc-29383/v) and co-cultured with MSC cells as described above. α-SMA gene expression was quantified with RT-PCR.

MSC treated with Adam8 immunodepletion medium: The human Adam8 antibody (R&D Systems, Minneapolis, MN, USA, AF1031) was used to immunodeplete the Adam8 soluble domain from the co-culture medium of MDA-MB-231 and MSC (48 h) and was then applied to MSC cells for culturing at different time points. Adam8 antibody (1:100 dilution) or goat IgG control were added to the collected co-culture medium for overnight incubation at room temperature, and protein A-agarose was added and incubated for 2 h on a roller system at 4 °C. The supernatants were collected and used to treat MSC. The total RNA was harvested from MSC cells at different time points, and gene expression of α-SMA/Ten-C/Vim was quantified using RT-PCR as described previously [13].

In vitro recombinant human Adam8 soluble domain activation and metalloproteinase activity assay: Recombinant human Adam8 soluble domain (Met1-Pro497) protein and its fluorogenic substrate(13aa) were ordered from R&D systems (Minneapolis, MN, USA). The Adam8 soluble domain activation and metalloproteinase assay was performed as per the manufacturer’s protocol. Briefly, recombinant human Adam8 soluble domain (Met1-Pro497) protein was diluted to 400 μg/mL in TCN assay buffer (50 mM Tris, 10 mM CaCL2, 150 mM NaCL, and pH 7.5). After adding an equal volume of 1.5 μg/mL thermolysin and incubation at 37 °C for 30 min, the reaction was stopped by adding Phosphoramidon to a final concentration of 0.05 mM at room temperature for 15 min. The activated recombinant human Adam8 soluble domain protein was diluted to 40 ng/μL in TCN assay buffer in the presence or absence of Adam8 aptamers at different concentrations and mixed for 1 min. The fluorogenic substrate was added at 37 °C and quantified with Cytation 1 (excitation: 320 nm; emission: 405 nm).

In vitro binding confirmation of Adam8 extracellular domain and Apt1 via binding competition assay: This protocol has been described previously [12]. Briefly, Ni-NTA-coated 96-well plates were coated with activated recombinant human Adam8 soluble domain his-tag protein (Ile17-Pro497, Acro Biosystems, Newark, DE, USA). Cy3-labeled Adam8-Apt1-26nt was synthesized via IDT (Integrated DNA Technologies, Coralville, IA, USA). A total of 10 pmol of Cy3-labeled Adam8-Apt1-26nt with or without 2 nmol of un-labeled Adam8-Apt1-26nt in 50 μL PBS was added to the above wells. After binding for 1 h and 3 washes with PBS, Cy3 intensity in each well was quantified using Cytation 1 (BioTek, Santa Clara, CA, USA).

In vivo pharmacokinetics (PK), intravenous vs subcutaneous (IV vs. SC) injection, and in vivo half-life quantification: 6-week-old female NOD SCID mice were used. The dosing schedules are listed below [Table 1]. Adam8 aptamer concentration was quantified using RT-PCR.

Evaluation of aptamer intracellular uptake: 4 × 10^5^ MDA-MB-231 cells, HepG2 cells, or MSC cells were seeded on 12-well plates to perform transfection with 80 μmol/L Cy3-labeled Adam8 aptamers and OptiMEM medium or with Lipofectamine 2000 packed aptamer and OptiMEM-positive controls. Images were taken at 72 h using a Leica SP2 confocal microscope.

Tumorsphere formation assay: A detailed protocol conducted in accordance with the Millipore Sigma manual was used, which has been described previously [14]. Briefly, the cancer cells were trypsinized into the single-cell suspension with Trypsin-EDTA (Sigma, St. Louis, MO, USA, T3924) for 2–4 min at room temperature, pipetted up and down 20 times using 1 ml tip, and two volumes of trypsin inhibitor solution (Sigma, T6414) were added to stop trypsin activity. The single-cell suspension was plated at 200 cells per cm^2^ with 3D tumorsphere Medium XF (Sigma, C-280700) on Corning Costar ultra-low attachment 6-well plate (Sigma, CLS3471). Equal amounts of MSC were added to the 6 well-plate insert (0.4 μm) and placed on the top. Adam8-Apt-1-26nt (3 μM) was added to the medium at 12 h, 48 h, and 72 h co-culture time points, respectively (daily). On day 7, images were acquired using Zeiss Apo Tome 2 microscope (10 × 20).

Xenograft regression model: All animal-handling activities and other procedures were approved by the University of South Florida Animal Care and Use Committee. A total of 1 × 10^6^ tumor cells w/wo MSC cells mixed with 50% Matrigel were implanted into 6-week-old female NOD SCID mice (Charles River, Wilmington, MA, USA) at the R4 location on their mammary fat pads. Bioluminescence imaging was performed weekly using an IVIS 100 imaging system (Xenogen, Hopkinton, MA, USA). Total photon counts (1 min) or counts/second were obtained. Two weeks after the cell implantation procedure, the mice were treated through tail vein injection with aptamer or saline control every two days until eight weeks had passed.

Fluorescence-activated cell sorting: Fresh primary tumors were obtained. Single-cell suspensions were prepared as reported previously [2]. The tissues were finely minced with surgical scissors and transferred to 10 mL collagenase–PBS solution (1 × PBS, PH7.4; 0.025% collagenase, 0.05% pronase, and 0.04% DNase I). After 1 h incubation at 37 °C, the tissue pellets were centrifuged at 300 g for 10 min at 4 °C and washed three times with 5 mL of PBS. The tissue homogenate was gently passed through a 70 μm pore nylon mesh filter at 4 °C. Cells were sorted using BD FACSMELODY (BD Biosciences, Franklin Lakes, NJ, USA). For GFP-positive cells sorting, cells were excited using a 488 nm laser, with emission data collected through a 530/30 band-pass filter. For RFP-positive cell sorting, cells were excited using a 561 nm laser, with emission data collected through a 610/20 band-pass filter. For BFP-positive cell sorting, cells were excited using a 405 nm laser, with emission data collected through a 440/50 band-pass filter. The sorted cells were collected in PBS and stored at −80 °C [15].

For RT-qPCR, GAPDH was used as the endogenous housekeeping gene, and delta-delta Ct analysis was performed. Pharmacokinetic and pharmacodynamic calculations were performed using GraphPad Prism version 9.1.1 (226) software (San Diego, CA, USA).

## 3. Results

### 3.1. Adam8, CA12, and CDH6 Selection

We have previously found that myCAF-mediated cancer stemness is required for the maintenance of the myCAF phenotype in this system [16]. In the results of the current series of RNAseq analyses [Figure S2], Adam8, CA12, and CDH6 were identified as candidates for myCAF-induced cancer-stemness-related genes with secreted proteins. siRNA constructs targeting each candidate were transfected into MDA-MB-231 and then cocultured with MSCs. In comparison to parental MDA-MB-231 + MSC cocultures, shRNA directed at CA12 and CDH6 did not significantly alter myCAF marker expression, α-smooth muscle actin (α-SMA), Vimentin (Vim), or Tenascin-C (TNC) [Figure S3]. Adam8-shRNA-transfected MDA-MB-231 cells did, however, result in the ablation of myCAF marker expression, indicating that Adam8 expression in cancer cells is required for the MSC development of the myCAF phenotype (Supplemental Data; Figure S1). We then proceeded to develop an aptamer against Adam8.

### 3.2. Synthesis and Characterization of Aptamer Targeting Adam8

**Figure 1:**
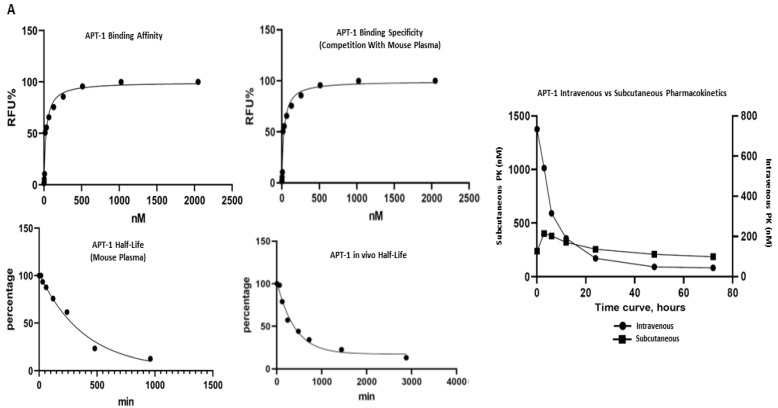

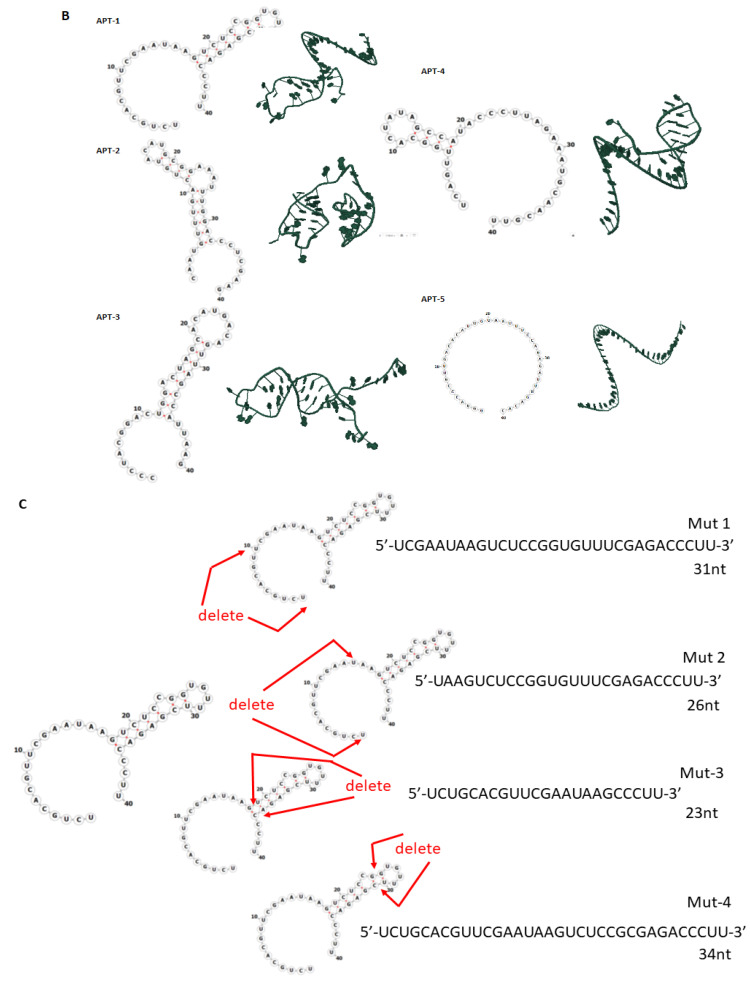

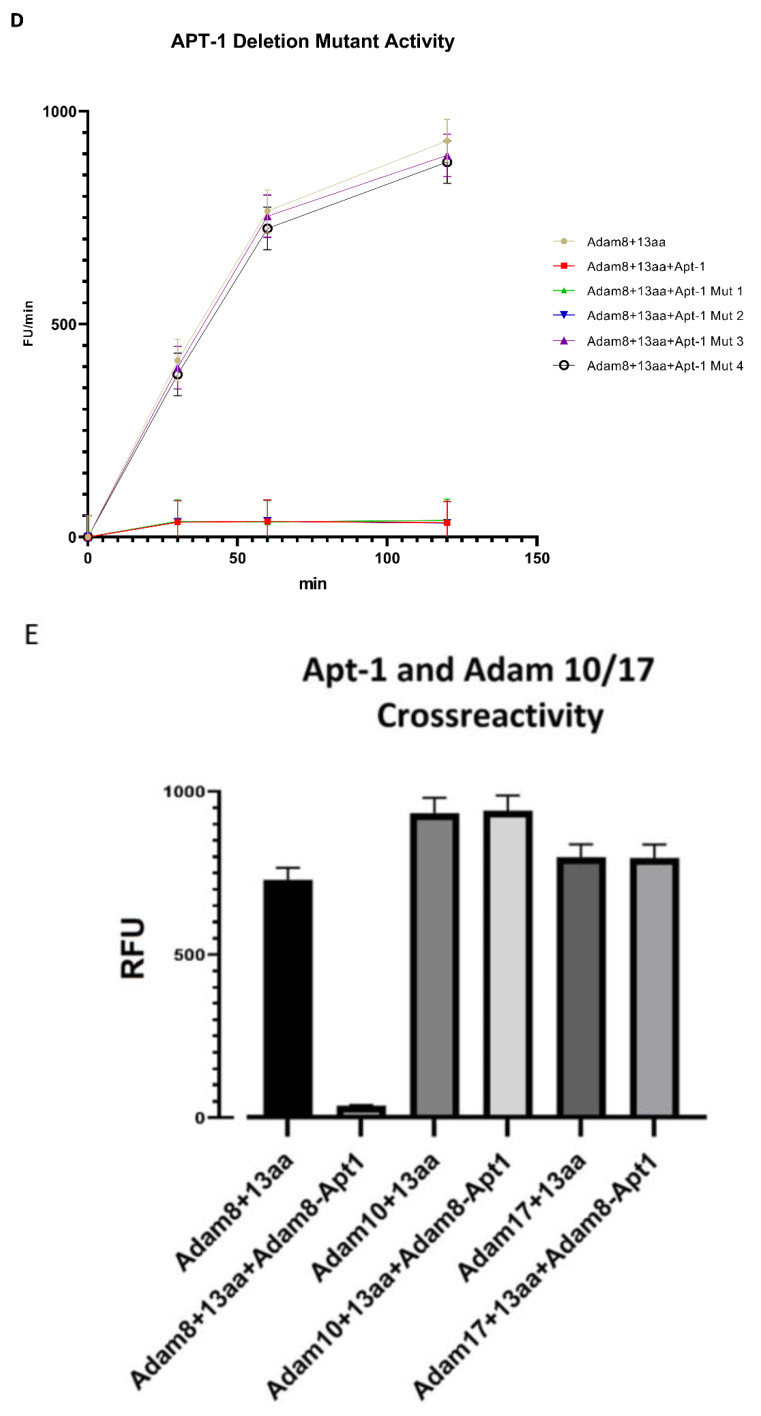

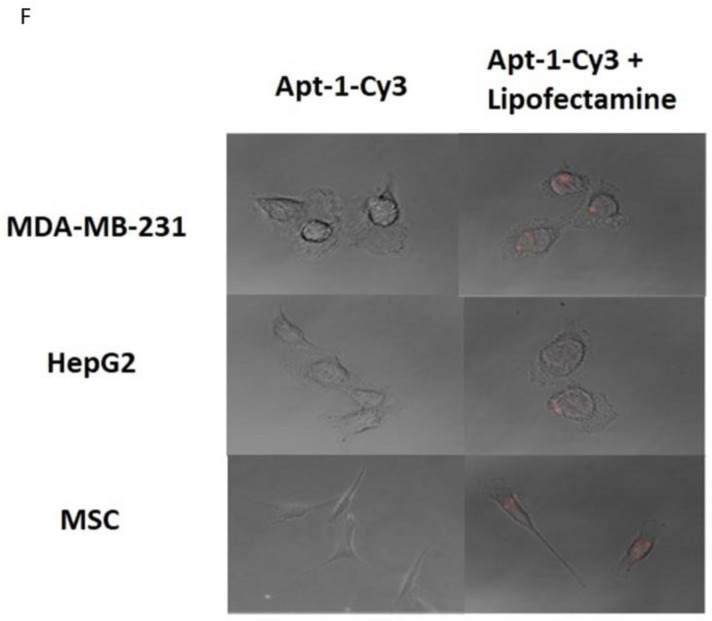
Synthesis and characterization of aptamer Apt-1 targeting Adam8. (**A**) Apt-1 binding affinity and specificity, in vivo and in vitro half-life, and intravenous and subcutaneous administration kinetics. (**B**) Candidate aptamers (Apt-1 through 5) targeting Adam8 sequences and theoretic tertiary structure. (**C**) Apt-1 deletion mutant constructs (Mut 1 through 4). (**D**) Apt-1 deletion mutant activities (n = 3). (**E**) Apt-1 cross-reactivity with Adam10 and Adam17 (n = 3). (**F**) Apt-1 is extracellular in the presence of MDA-MB-231 and HepG2 cells (20 × 20).

Thirty clones were sequenced following the sixth round of SELEX, eleven of which were the Adam8 aptamer (Apt-1) [Figure S4]. Pharmacodynamic and pharmacokinetic analyses were performed. The Kd values of Apt-1 and four other clones were determined; the Kd value of Apt-1 was the lowest at 29.7 nmol/L (19.25–44.12, 95% CI) (Figure 1A). The in vitro half-life in mouse plasma was 267 min (174–471, 95% CI), while the in vivo half-life was 278 min (174–455, 95% CI). Pharmacokinetics for intravenous and subcutaneous delivery of Apt-1 demonstrated stable (82 nM and 99 nM, respectively) serum concentrations up to 72 h. The predicted secondary structure of Apt-1 contains the usual stem–loop structure of RNA aptamers and is shown in Figure 1B along with those of APT-2, 3, 4, and 5. The mutagenesis of Apt-1 was then induced to determine the active site (Figure 1C). Deletion constructs were then tested using in vitro recombinant human Adam8 soluble domain activation and metalloproteinase activity assays. The results demonstrate that only Apt-1 retained active (Figure 1D). Adam10 and Adam17 cross-reactivity with Apt-1 was determined (Figure 1E). The coincubation of Apt-1 with Adam10 or Adam17 in the presence of the 13aa substrate did not impact Adam10 and Adam17 metalloproteinase activity. Apt-1 was linked to Cy3 and incubated with MDA-MB231, HepG2, and MSC in the presence and absence of lipofectamine (Figure 1F) to selectively block Adam8 metalloproteinase activity.

### 3.3. In Vitro Activity of Apt-1

**Figure 2:**
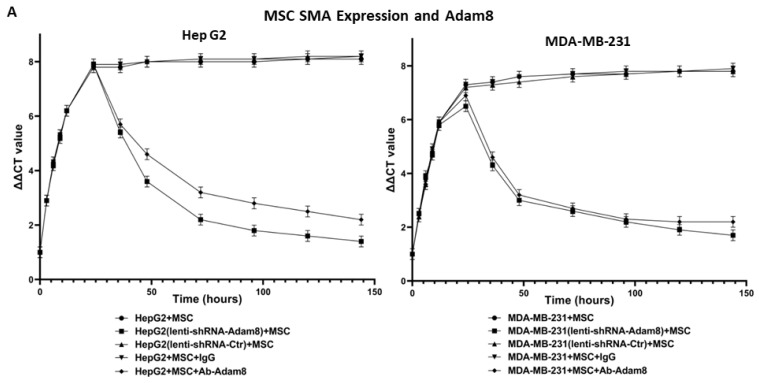

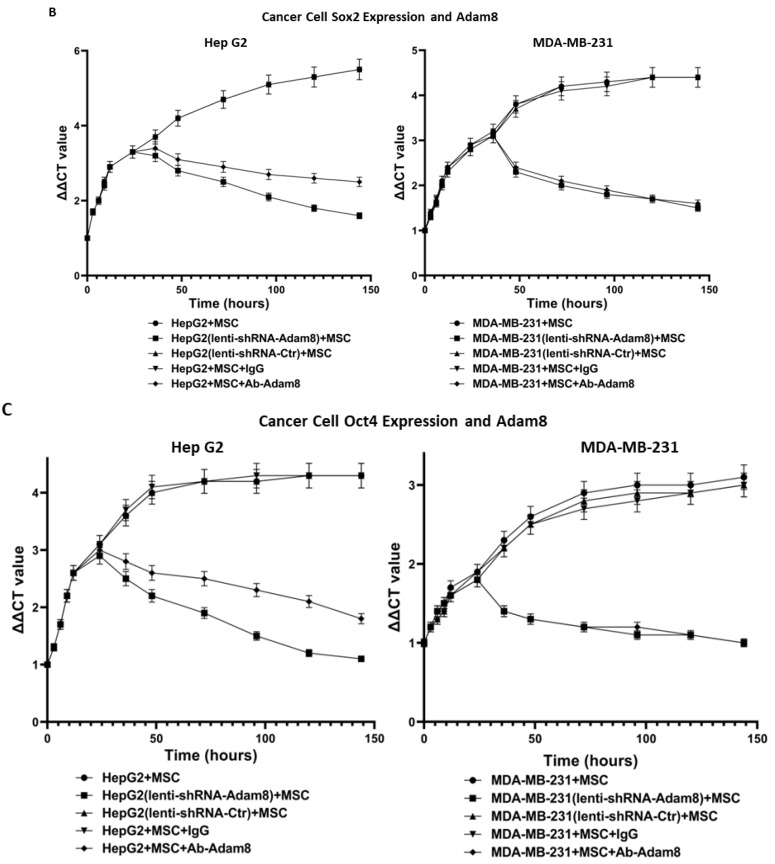

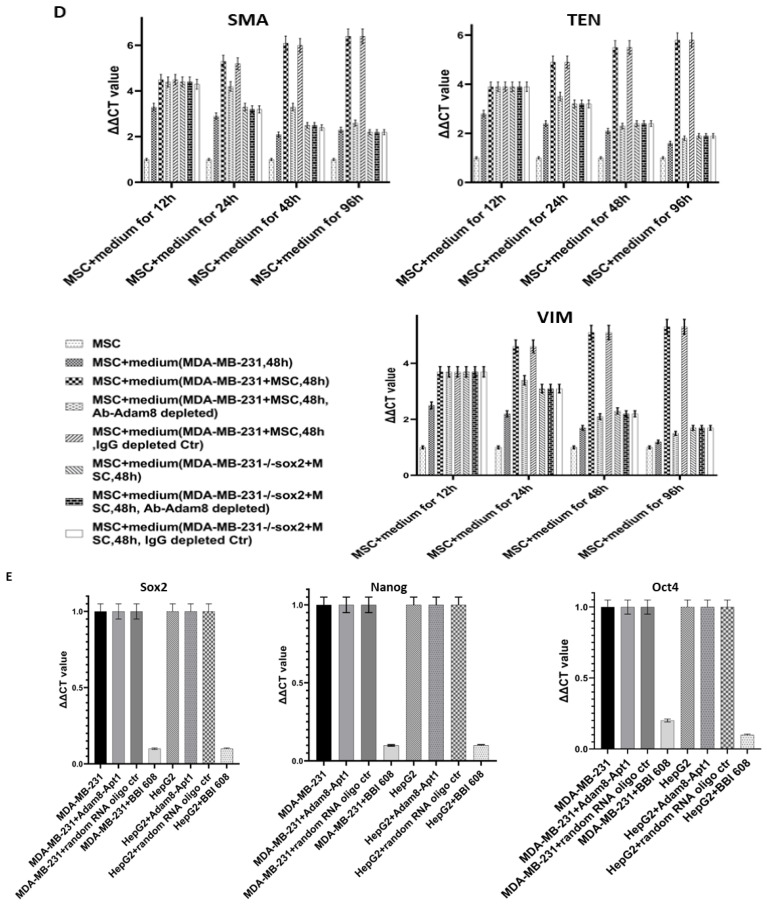
Effect of Adam8 and cancer stemness on the myCAF phenotype. (**A**) Adam8 ablation effect on time-dependent myCAF expression of α-SMA in MDA-MB231 + MSC and HepG2 + MSC co-cultures (n = 3). (**B**) Effect of Adam8 ablation on time-dependent cancer cell expression of sox2 in MDA-MB231 + MSC and HepG2 + MSC co-cultures (n = 3). (**C**) Effect of Adam8 ablation on time-dependent cancer cell expression of Oct4 in MDA-MB231 + MSC and HepG2 + MSC co-cultures (n = 3). (**D**) Conditioned media studies examining myCAF marker expression and effect of cancer + MSC (and cancer (sox2-KD) + MSC) coculture media for which Adam8 has been depleted (n = 3). (**E**) Cancer stemness markers (sox2, Nanog, and Oct4) in MDA-MB-231 and HepG2 cocultures with MSC, Apt-1, and/or the stemness inhibitor BBI 608. The uncropped blots are shown in Figure S8.

To determine the potential role of Adam8 in the cell-signaling crosstalk between MSCs and cancer cells toward the induction of the myCAF phenotype with a concomitant increase in cancer stemness, we performed coculture studies in which human MSC cells were cultured with either MDA-MB-231 breast cancer cells or HepG2 liver cancer cells and the Adam8 antibody added at different time points to block Adam8 bioactivity. In selected instances, MSCs were cultured with MDA-MB-231 (Adam8-KD) or HepG2 (Adam8-KD), and in others, Adam8 mAb was added (Figure 2A). After coculturing for periods of up to 144 h, MSC α-SMA expression was measured via real-time PCR as a reflection of the myofibroblast myCAF phenotype (MDA-MB-231 alone or HepG2 alone were used as reference samples). In all instances, the coincubation of cancer cells with MSCs resulted in increased α-SMA expression with a plateau at 30 h, except in the presence of Adam8 mAb or lentivirus Adam8 KO, in which there was a progressive decline in α-SMA expression starting at approx. 30 h and ranging to a level 1.5–2-fold greater than the baseline. We also determined the HepG2 and MDA-MB-231 expression of the cancer stemness markers sox2 and Oct4 in the coculture models (Figure 2B,C). In a manner similar to that presented for α-SMA, the cancer cell expression of both stemness markers increased with a plateau at 30 h, except in instances wherein Adam8 mAb or lentivirus Adam8 KO were present. These results indicate that Adam8 is required for the maintenance of the myCAF and cancer stemness phenotype in these coculture models.

Conditioned media studies were then performed. MSCs were cocultured alone, with MDA-MB-231, or with MDA-MB-231 (sox2-KD) for 48 h. In selected instances, the medium was supplemented with Adam8 mAb to generate Adam8-depleted media or an IgG control. The media were then transferred to MSCs, and myCAF markers were determined after 12, 24, 48, and 96 h (Figure 2D). In the MSCs exposed to the MDA-MB231 medium, the levels of myCAF markers peaked at 12 h, with a progressive decline thereafter. In contrast, MSCs exposed to the MSC + MDA-MB-231-primed medium expressed myCAF markers in a progressively increasing manner with a near plateau at 96 h. This pattern was ablated in the MSCs exposed to the MSC + MDA-MB-231 medium for which Adam8 had been depleted, presenting a myCAF expression pattern similar to that of the MDA-MB-231 media. In MSC exposed to media from MSC + MDA-MB-231 (sox2-KD), myCAF markers peaked at 12 h with progressive decline afterwards regardless of the presence or absence of Adam8. As controls, MDA-MB-231 or HepG2 were cultured with Apt-1 and/or the pharmacologic stemness inhibitor BBI 608 (Figure 2E). In this setting, Apt-1 does not directly alter cancer stemness marker expression. These results suggest that Adam8 in the extracellular space (and cancer stemness) are required for the maintenance of the myCAF phenotype following initial induction.

**Figure 3:**
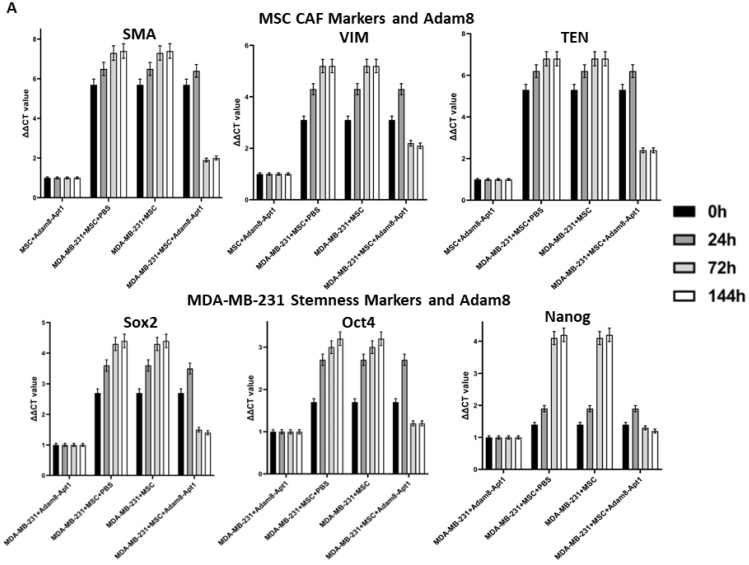

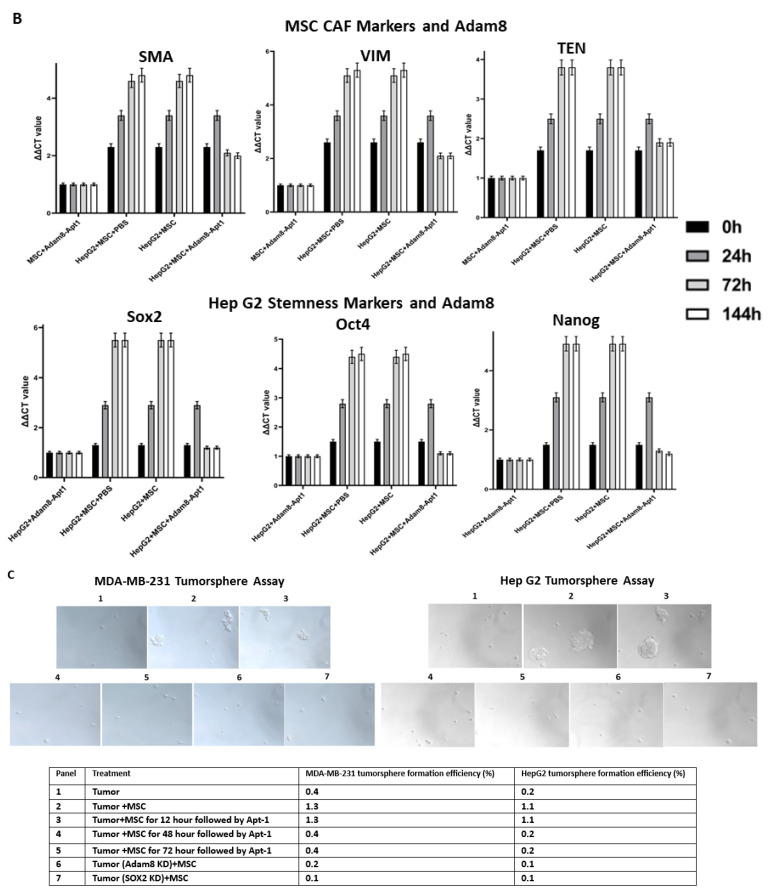

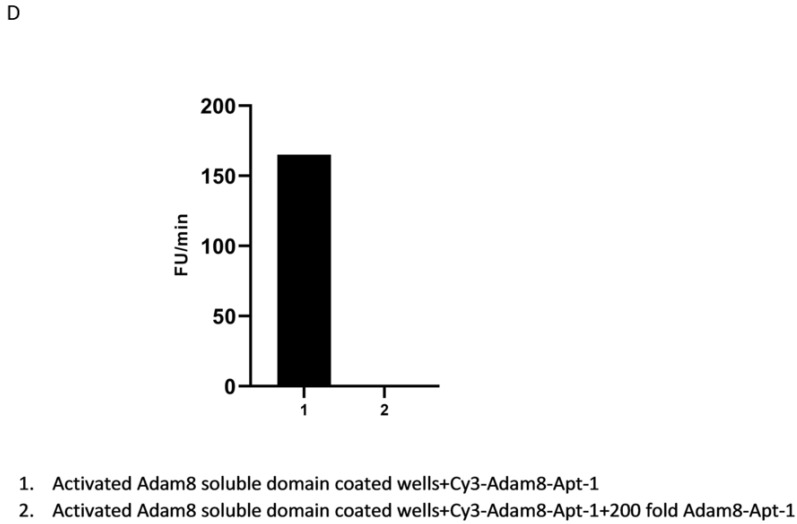
Time-dependent effect of Apt-1 on myCAF and cancer stemness markers. (**A**) Time-dependent effect of Apt-1 on myCAF and cancer stemness markers in MDA-MB-231 + MSC cocultures (n = 3). (**B**) Time-dependent effect of Apt-1 on myCAF and cancer stemness markers in HepG2 + MSC cocultures (n = 3). (**C**) Tumorsphere assay for MDA-MB-231 and HepG2 cocultures with MSC and Apt-1(10 × 20). (**D**) Apt-1 pulldown studies performed based on binding competition with 200-fold excess non-labeled Apt-1.

Apt-1 activity was then assessed using the same coculture system (Figure 3A,B). In these studies, the levels of myCAF phenotype markers, α-SMA, TenC and Vim, and the cancer stemness markers sox2, Oct4, and Nanog were measured in the MSCs and cancer cells. While the levels of both myCAF and stemness markers were increased following coculture, the administration of Apt-1 was associated with lowered expression of both after a peak at 24 h in both MDA-MB-231 and HepG2 cells. To functionally confirm Apt-1 inhibition of cancer cell stemness, we performed a tumorsphere formation assay with MDA-MB-231 or HepG2 cells co-cultured with MSCs (Figure 3C). Apt-1 treatment at 48 h and 72 h co-culture time points significantly decreased tumorsphere formation efficiency. These results indicate that Apt-1 blocked the maintenance of the myCAF and cancer stemness phenotypes in the coculture models [Figures S6 and S7].

Apt-1 pulldown studies were performed based on binding competition. The His-tag-labeled activated human Adam8 soluble domain was coated on Ni-NTA-96-well plates and incubated with Cy3-labeled Apt-1 only or with a His-tag-labeled and -non-labeled Apt-1 mixture (1:200) to determine the degree of binding competition. The results showed that Cy3 intensity was completely abolished in the Apt-1 mixture binding group (Figure 3D). We also performed in vitro Adam8 soluble domain metalloproteinase activity assays. After applying an internally quenched, MCA/Dpa-labeled, Adam8 fluorogenic substrate (13aa) with or without the addition of Apt-1 or Apt-1 deletion mutants, the results indicate that Apt-1 can effectively inhibit human Adam8 soluble domain metalloproteinase in vitro. Overall, these studies confirmed Apt-1’s binding to the shed extracellular soluble Adam8 and the inactivation of its metalloproteinase activity.

### 3.4. In Vivo Activity of Apt-1 against Established Tumor

**Figure 4:**
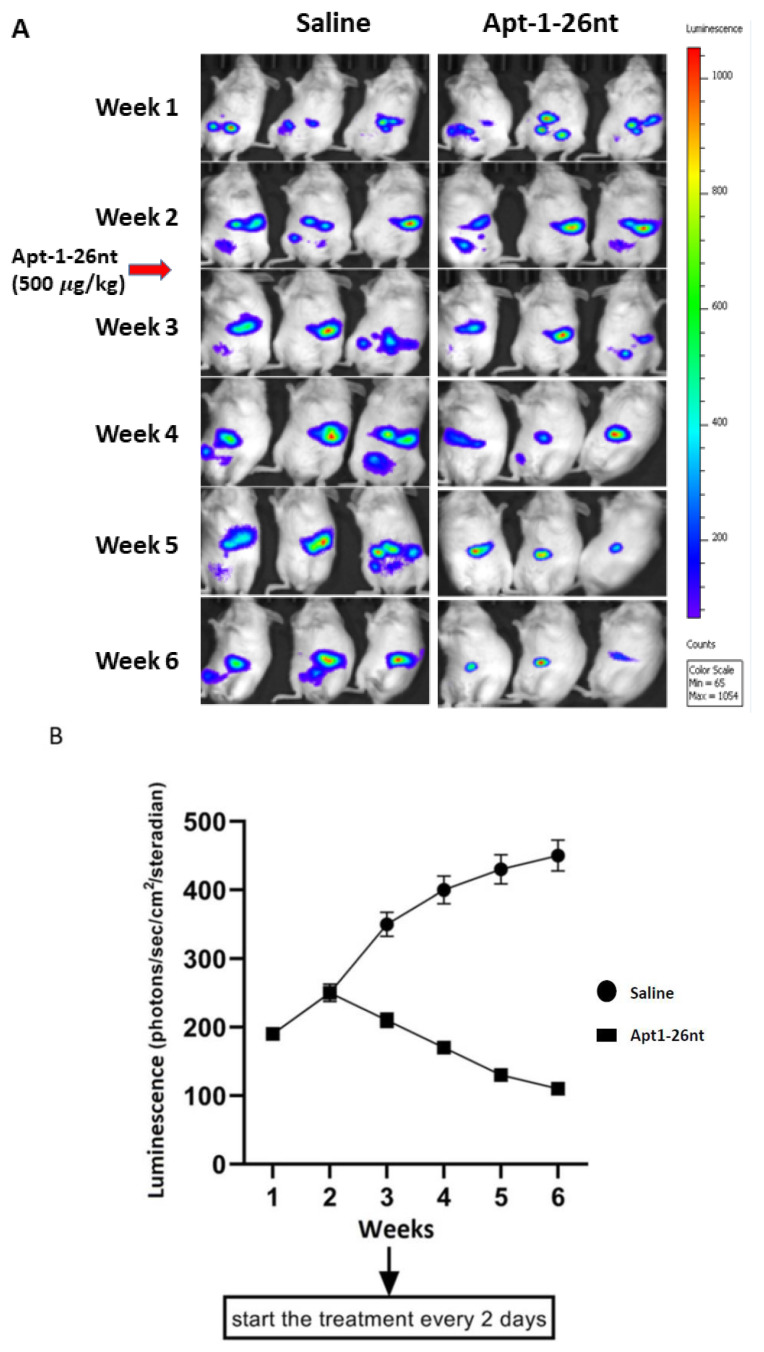

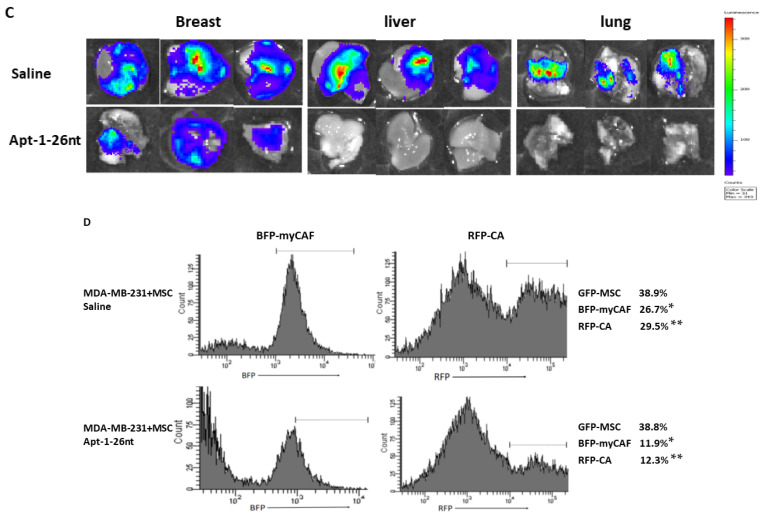
MDA-MB-231 + MSC xenografts in NOD-scid mice. (**A**) Time course of MDA-MB-231 luciferase activity in saline- and Apt-1-26nt-treated mice. (**B**) Time course of MDA-MB-231 luminescence activity in saline- and Apt-1-26nt-treated mice (n = 3). (**C**) Breast, lung, and liver MDA-MB-231 luciferase activity in saline- and Apt-1-26nt-treated mice after sacrifice at week 6. (**D**) Flow cytometry regarding RFP-MDA-MB-231, GFP-MSC, and α-SMA inducible BFP-myCAF in saline- and Apt-1-26nt-treated mice after sacrifice at week 6. ** and * indicate *t*-test *p*-values < 0.05.

We then tested the efficacy of Apt-1-26nt in an in vivo NOD-scid mouse model of human breast cancer (Figure 4). The R4 mammary fat pads of 6-week-old female NOD SCID mice were injected with 106 MDA-MB-231 cells expressing luciferase/RFP along with MSCs expressing GFP and α-SMA promoter-inducible BFP. Three mice were used per group. Three weeks after inoculation, treatment was initiated with Adam8 Apt-1-26nt (500 μg/kg or saline control via tail vein injection every 2 days). The images demonstrate that Apt-1-26nt administration is associated with the stabilization of tumors with minimal growth (Figure 4A). In contrast, the cocultures of MDA-MB-231 cells (Adam8-KD) with MSC were not sustained beyond the first week. Luciferase activity corroborated the observation that Apt-1-26nt stabilized and/or decreased tumor growth (Figure 4B). Following sacrifice at 6 weeks, the primary tumors, livers, and lungs were examined with respect to luciferase activity. These images indicate that there were no tumor metastases in the livers and lungs of the Apt-1-26nt-treated animals (Figure 4C). Flow cytometry was performed on the explanted tumors (Figure 4D). The Apt-1-26nt-treated animals exhibited significantly fewer myCAF and cancer cells compared to the saline-treated controls. These data suggest that Apt-1-26nt inhibits the growth of established tumor cells in vivo in parallel with decreased amounts of myCAF.

## 4. Discussion

In this paper, we characterized a novel RNA aptamer, Apt-1, directed against the extracellular Adam8 soluble metalloproteinase domain. In MSC co-culture studies employing MDA-MB-231 breast cancer cells and HepG2 liver cancer cells, Apt-1 inhibits MSCs’ adoption of the myCAF phenotype and cancer cell stemness. In an in vivo murine xenotransplant model, Apt-1 inhibited MDA-MB-231 breast cancer growth and metastasis while also decreasing the expression of markers for the myCAF phenotype and cancer stemness. These results suggest that soluble Adam8 is a potential drug target and that Apt-1 is a novel agent directed against the Adam8 soluble metalloproteinase fragment.

RNA aptamers represent a novel category of therapeutic agents [8,9,17]. They are 12–80 nt ss RNA oligonucleotides with stable three-dimensional conformations that tightly and specifically bind to their target proteins. RNA aptamers bind to extracellular targets, such as the soluble Adam8 metalloproteinase domain. As demonstrated by Apt-1 and OPN-R3, these RNA aptamers typically exhibit binding affinities in the low nanomolar to picomolar range, are heat stable, are not immunogenic, and exhibit minimal batch-to-batch variability [Figure S5]. Modifications, such as amino- or fluoro-substitutions at the 2′ position of pyrimidines, reduce the degradation of aptamers by nucleases. The biodistribution and clearance of aptamers are altered by the addition of polyethylene glycol and cholesterol. In addition, SELEX allows for selection from libraries consisting of up to 1015 ligands in order to generate high-affinity oligonucleotide ligands capable of binding to purified biochemical targets such as Adam8.

The potential underlying impediments to the clinical utility of aptamers include their susceptibility to nuclease degradation, renal filtration, and excretion; the potential for immunogenicity; and their assumption of altered in vivo structures that results in decreased function.

A recent search of ClinicalTrials.gov for the term “aptamer” yielded 53 current and past trials in which aptamers were tested as biosensor, imaging, or therapeutic agents for a variety of pathologies including bladder CA, COVID-19, HIV, age-related macular degeneration, CD30+ lymphoma and solid tumors, and metastatic colorectal and pancreatic cancers. Specifically in the realm of cancer therapeutics, two aptamers, namely, AS1411 and NOX-A12, have undergone clinical trials [18]. AS1411 is the first aptamer for the treatment of cancer in clinical application. In a phase I clinical trial, 17 patients with renal and non-small cell lung cancers with advanced solid tumors were treated with AS1411 [19]. In the corresponding phase II trial, AS1411 was administered to 35 patients with metastatic renal cell carcinoma. The conclusion of these trials was that the anti-cancer effects of AS1411 were minor and that the toxicity profile was acceptable [20]. NOX-A12 is a pegylated L-type RNA aptamer resistant to nuclease degradation that binds to chemokine CXCL12, which plays an important role in the TMEN and cancer cell signaling [21]. In the phase I/II clinical trials, 28 patients with CLL were treated with a combination therapy including NOX-A12. Consequently, 86% of patients had an overall response to treatment with a median progression-free survival of 15.4 months. No additional toxicity was associated with NOX-A12 [22].

Adam proteases are a group of membrane-bound enzymes with “sheddase” functions [23,24]. Soluble ectodomain shedding of membrane proteins is an integral part of cell signaling in multiple settings, including cancer [1]. Protumorigenic effects have been associated with essential (Adam 10 and 17) and inducible proteases (Adam 8, 9, 12, 15, and 19). Adam8 was first identified in monocytic immune cells and subsequently demonstrated to be selectively expressed [23]. Adam8 was initially thought to be immune-specific as the result of its induction via inflammatory signaling, including by tumor necrosis factor, lipopolysaccharide, interleukin-1, and interferon-γ. Studies in Adam8 KO mice indicate that Adam8 is not required for normal development and homeostasis [25].

Increased expression of Adam8 has been correlated with enhanced tumor growth and metastasis in breast, brain, pancreatic, liver, colon, and kidney cancers [1]. However, the role of Adam8 in cancer has not been well characterized. Adam8 is highly expressed in breast tumors, which is associated with an aggressive phenotype and poor patient outcomes [26,27]. In primary breast tumors, Adam8-positive cells are most common in the invasion zone; Adam8 expression is maintained with metastases. Previous studies incorporating MDA-MB-231 Adam8 KO mouse xenograft models showed the presence of significantly smaller tumors, decreased levels of circulating tumor cells, and lower numbers of brain metastases [26]. In hepatocellular carcinoma, high Adam8 expression is found in the majority of cases. Elevated Adam8 levels are associated with increased serum Alpha-fetoprotein (AFP), advanced tumor stage, poor differentiation, increased tumor recurrence and metastasis, and reduced survival [28,29]. Adam8 KO in HepG2 cells exhibited reduced cell migration and invasion. Orthotopic murine xenograft models with Adam8 KO HepG2 presented significantly smaller tumors. Monoclonal antibody directed against Adam8 improved survival and reduced loss of body weight [30]. Adam8 mAb also lowered AFP; slowed the progression of HCC; induced the expression of Casp3, Bax, and P53; and inhibited the expression of VEGF-A, PCNA, and Bcl2 in mouse livers. Adam8 has not been previously linked to the maintenance of the myCAF phenotype in the TMEs of breast or liver cancer.

Cancer growth and metastasis are regulated by reciprocal cross talk between the tumor microenvironment (TME) and cancer stem cells. The TME consists of highly complex and dynamic molecules, blood vessels, and various cells, which surround cancer cells. As one key TME component, myCAF carries out multiple functions in order to manipulate cancer development, such as facilitating extracellular matrix remodeling, accelerating angiogenesis, promoting cancer cells’ epithelial–mesenchymal transition, increasing cancer cell invasion and metastasis, and facilitating the evasion of tumor immunosurveillance and therapeutic resistance. Although myCAF has been classified into subtypes based on cell surface markers or transcriptome profiling, there is no consensus regarding myCAF subtype classification and the subtypes’ identification markers, so the most typical intracellular markers of tumor-promoting myCAF cells are α-SMA, Vim, and TNC. Cancer stem cells are functionally characterized by self-renewal and differentiation, which reprograms the TME to favor tumor initiation, heterogeneity, immune escape, invasion, metastasis, and therapeutic resistance. There is not a consensus marker for cancer stem cells (CSC); therefore, several pluripotent stem cell transcription factors, such as sox2, Oct4, and Nanog, are commonly applied to measure cancer stemness [31].

Molecular drivers originating from the TME–CSC interaction network are ideal targets either in diagnostic or therapeutic clinical practice. Our lab previously found that cancer-cell-derived osteopontin (OPN), a matricellular protein, promotes bone-marrow-derived mesenchymal stem cells’ (MSCs) resident transformation into myCAF, while maintenance is feedback-regulated by CSC stemness. In this study, we identified Adam8 as a sox2-dependent protein expressed in MDA-MB-231 breast cancer cells when cocultured with MSCs. We previously found that myCAF-induced cancer stemness is required for the maintenance of the myCAF phenotype, suggesting that the initiation and maintenance of the myCAF phenotype required distinct cell-signaling crosstalk pathways between cancer cells and myCAF [16]. Our strategy was to isolate the cancer genes upregulated in the MSC coculture and downregulated in cancer (sox2-KD) when similarly cocultured with MSC. Adam8 was then identified as a candidate secreted protein induced by myCAF-mediated cancer stemness. Adam8 has a known sheddase function against which we developed an RNA aptamer, namely, Apt-1. The Apt-1-mediated blockade of the extracellular soluble Adam8 metalloproteinase domain abolishes the previously initiated myCAF phenotype (blocks the maintenance of the myCAF phenotype). In addition, cancer stemness is significantly decreased as a result, although previous studies have demonstrated that Apt-1 does not directly alter cancer stemness [16]. Xenograft models show that Apt-1 administration is associated with decreased tumor growth and metastasis, while flow cytometric analyses demonstrate significantly decreased fractions of myCAFs with Apt-1. The role of soluble Adam8 in the maintenance of the myCAF phenotype has not been previously characterized. Parenthetically, this study also suggests that the induction or initiation of the myCAF phenotype may be distinct from the maintenance of the myCAF phenotype. Additional studies directed toward identifying the substrate or receptor target of the soluble Adam8 fragment responsible for the chronic expression of the myCAF phenotype are underway.

## 5. Conclusions

The signal pathways that initiate the development of the myCAF phenotype may be distinct from those required for myCAF maintenance post-initiation. Extracellular Adam8 sheddase activity is required for the maintenance of the OPN-induced myCAF phenotype, and this aptamer, Apt1-, may serve as a vehicle for the further investigation of this new pathway.

## Figures and Tables

**Table 1:** Dosing schedules.

	PK	IV vs. SC	In Vitro Half-Life
injection location	tail vein	neck	0.5 pg + 50 μL mouse plasma incubation at 37 °C in 5% CO_2_
aptamer amount	2.2 nM	2.2 nM	
blood harvest time points	3, 6, 12, 24, 48 h	3, 6, 12, 24, 48, 72 h	3, 6, 12, 24, 48 h

## Data Availability

The data presented in this study are available in this article and Supplementary Material.

## Supplementary Materials

The following supporting information can be downloaded at https://www.mdpi.com/article/10.3390/cancers15123254/s1, Figure S1: A-SMA gene expression in MSC cells co-cultured with MDA-MB-231-Adam8-KD; Figure S2: RNAseq data; Figure S3: CA12 and CDH6 knockdown in MDA-MB-231 cells. SMA/TNC/VIM gene expression was quantified using RT-PCR in MDA-MB-231-CA12-KD or CDH-KD co-cultured cells; Figure S4: Adam8 RNA aptamer sequences; Figure S5: Adam8 Apt-1-26nt aptamer does not induce immunogenicity; Figure S6: MDA-MB-231 cell invasion assay; Figure S7: MDA-MB-231 cell wound-healing assay. Figure S8: Adam8 Knockdown in MDA-MB-231 Cells.

## Abbreviations

α-SMA	alpha smooth muscle actin;
TenC	Tenascin C;
Vim	Vimentin;
sox2	SRY related HMG box transcription factor 2;
Oct4	Octamer binding transcription factor 4;
Nanog	Homeobox protein Nanog;
CA12	carbonic anhydrase 12;
CDH6	Cadherin 6.
Adam8-KD	Adam8 shRNA knockdown
Sox2-KD	Sox2 shRNA knockdown
Apt-1	5′-UCUGCACGUUCGAAUAAGUCUCCGGUGUUUCGAGACCCUU-3′
Apt1-1-26nt	5′-UAAGUCUCCGGUGUUUCGAGACCCUU-3′
