# Supplementary material for: RNA Aptamer Targeting of Adam8 in Cancer Growth and Metastasis
Source: Cancers (Basel). 2023 Jun 20;15(12):3254. doi: 10.3390/cancers15123254 (PMC10297143; doi:10.3390/cancers15123254)
Supplement: Supplementary file 1 [file cancers-15-03254-s001.zip › cancers-2392557-supplementary.pdf]

Supplementary results

Methods of Supplementary Results:

Mouse peripheral blood mononuclear cell (PBMC) isolation and Adam8-Apt1-26nt treatment: The Ficoll–Paque density gradient centrifugation-based method used to isolate PBMC was described previously (1). Briefly, 1ml of anticoagulant-treated mouse blood was mixed with the same volume of RPMI 1640 media. This diluted blood sample was loaded onto 3 ml of Ficoll–Paque media (1.076g/ml); then, it was centrifuged at 400g X 30 min at 20°C, the upper layer was carefully discarded, and the lower layer was transferred to a new tube. The cells were washed with three volumes of RPMI 1640 media and centrifuged at 400g X 10min at 20°C. The cell pellet was resuspended with RPMI 1640 containing 10% Fetal Bovine Serum and treated with 3uM Adam8-Apt1-26nt for 24h in 5% CO_2 incubator at 37°C. Both adherent and suspension cells were harvested, and total RNA isolation was performed.

Mouse bone marrow cells' isolation: mouse femur and tibia were dissected and flushed with RPMI 1640 media containing 10% fetal bovine serum. The flushed bone marrow cells were treated with 3uM Adam8-Apt1-26nt for 24h in a 5% CO_2 incubator at 37°C. Both adherent and suspension cells were harvested, and total RNA isolation was performed.

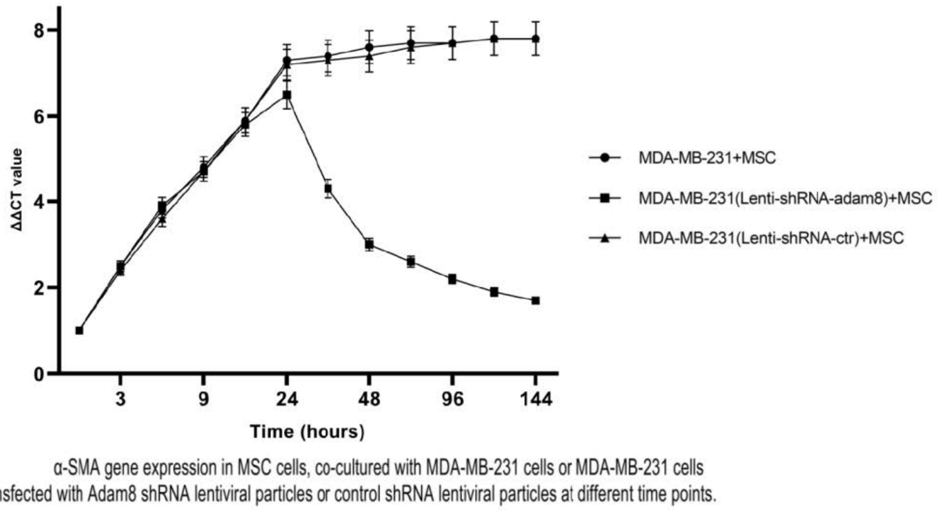

Figure S1. A-SMA gene expression in MSCs co-cultured with MDA-MB-231-Adam8-KD.

common gene list

ABCC2	MYL6P1	HIST1H2BN	RP11-159C21.4	RP3-445N2.1	SYT1
AC004656.1	ADAM8	ID1	RP11-161H23.5	RP3-508I15.18	TMEM189
AC005086.1	RNU1-106P	IL7R	RP11-1E11.1	RP3-510D11.1	TPRN
AC018630.1	AL139328.1	KIF17	RP11-215G15.4	RP4-553F4.6	ZNF33B
AC106753.1	ARC	KRTAP3-2	RP11-249L21.4	RP4-725G10.4	
ALG9	ARHGAP19-SLIT1	KRTAP3-3	RP11-264B14.2	RP5-902P8.10	
AC013403.13	BNIPL	LINC00106	RP11-282O18.7	SCARNA9	
CORO7-PAM16	CA12	LRRN4	RP11-295P9.3	SNORA34	
CTD-2410N18.5	CDH6	MARVELD3	RP11-33O4.1	SNORA45	
AC046143.3	CRYAA	MTND1P15	RP11-367G18.2	SNORA51	
CTD-307407.11	CTA-268H5.12	MT-ND4	RP11-426L16.8	SNORA72	
CTD-3214K23.1	CTC-425F1.2	REV3L-IT1	RP11-475J5.6	SNORD100	
AC073610.5	CTC-429P9.2	RHBDL1	RP11-500M8.7	SNORD104	
AC073958.2	CTC-457E21.7	RN7SL114P	RP11-697N18.1	SNORD12	
EIF1AX-AS1	CYP27B1	RN7SL624P	RP11-723D22.3	SNORD17	
AC091948.1	DUSP10	RN7SL694P	RP11-737O24.5	SNORD38A	
AC100830.4	ELAVL2	RN7SL811P	RP11-867G23.1	SNORD4A	
AC118344.1	FMN1	RNA5SP383	RP11-867G23.8	snoU13	
AC131012.1	FSBP	RP11-112J3.16	RP1-228P16.7	SPDYA	
MIR5010	HCG22	RP11-152F13.10	RP3-433F14.1	SUB1P3	

Figure S2. RNA seq data.

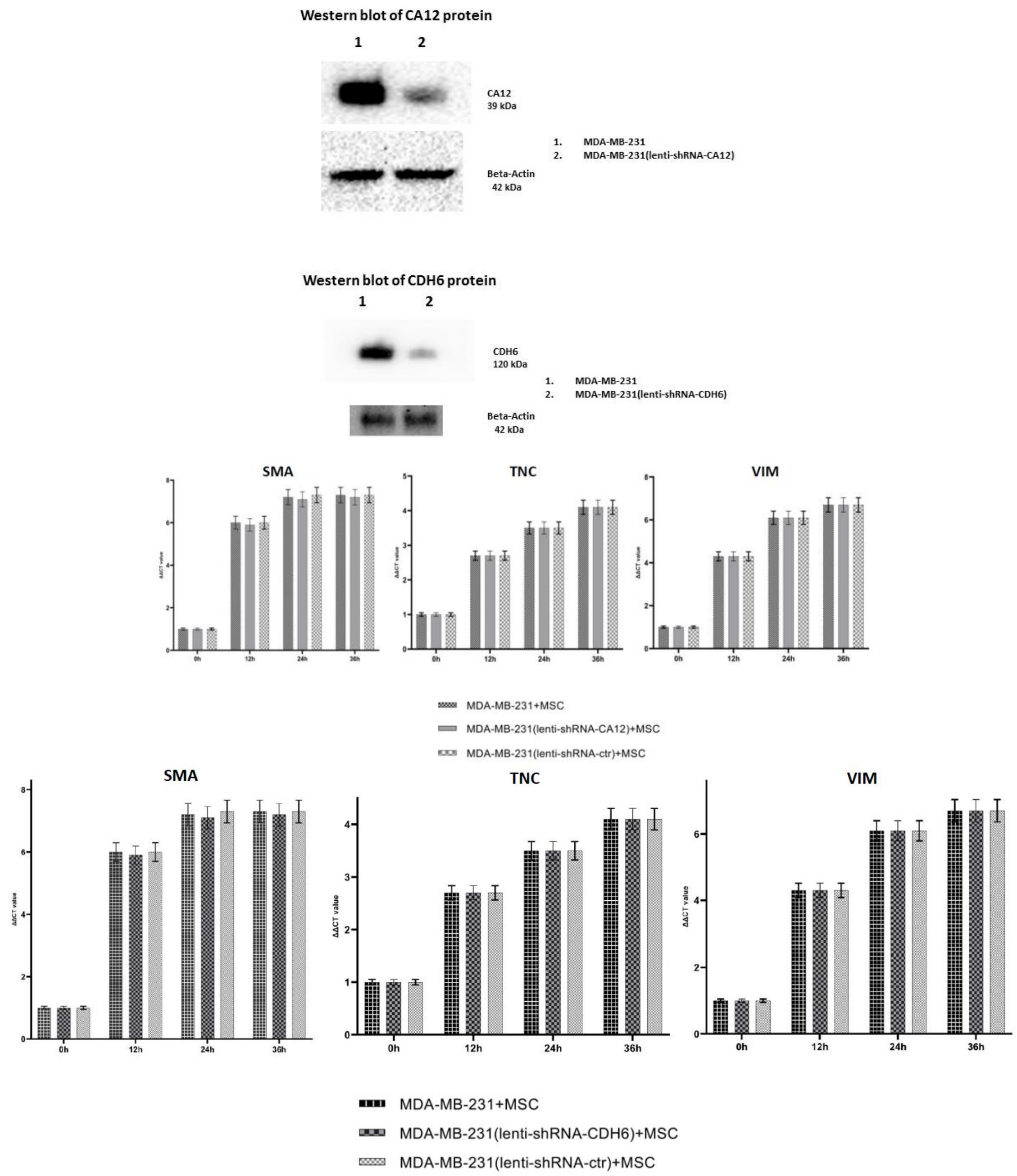

Figure S3. CA12 and CDH6 knockdown in MDA-MB-231 cells. SMA/TNC/VIM genes expression were quantified with RT-PCR in MDA-MB-231-CA12-KD or CDH-KD co-cultured cells.

APT-1: 5′-ucugcacguucgaauaagucuccgguguuucgagacccuu-3′
APT-2: 5′-caauguuugacuguacaugcggaaauuuggacccucgaag-3′
APT-3: 5′-cccuacggacuggacuagcacaugacaguuagccauuaag-3′
APT-4: 5′-ucaguuggcacuauagccauacccuuagaaaugcaacguu-3′
APT-5: 5′-gguacccguugacacauuguaauuuccagagauuugacac-3′

Figure S4. Adam8 RNA aptamer sequences.

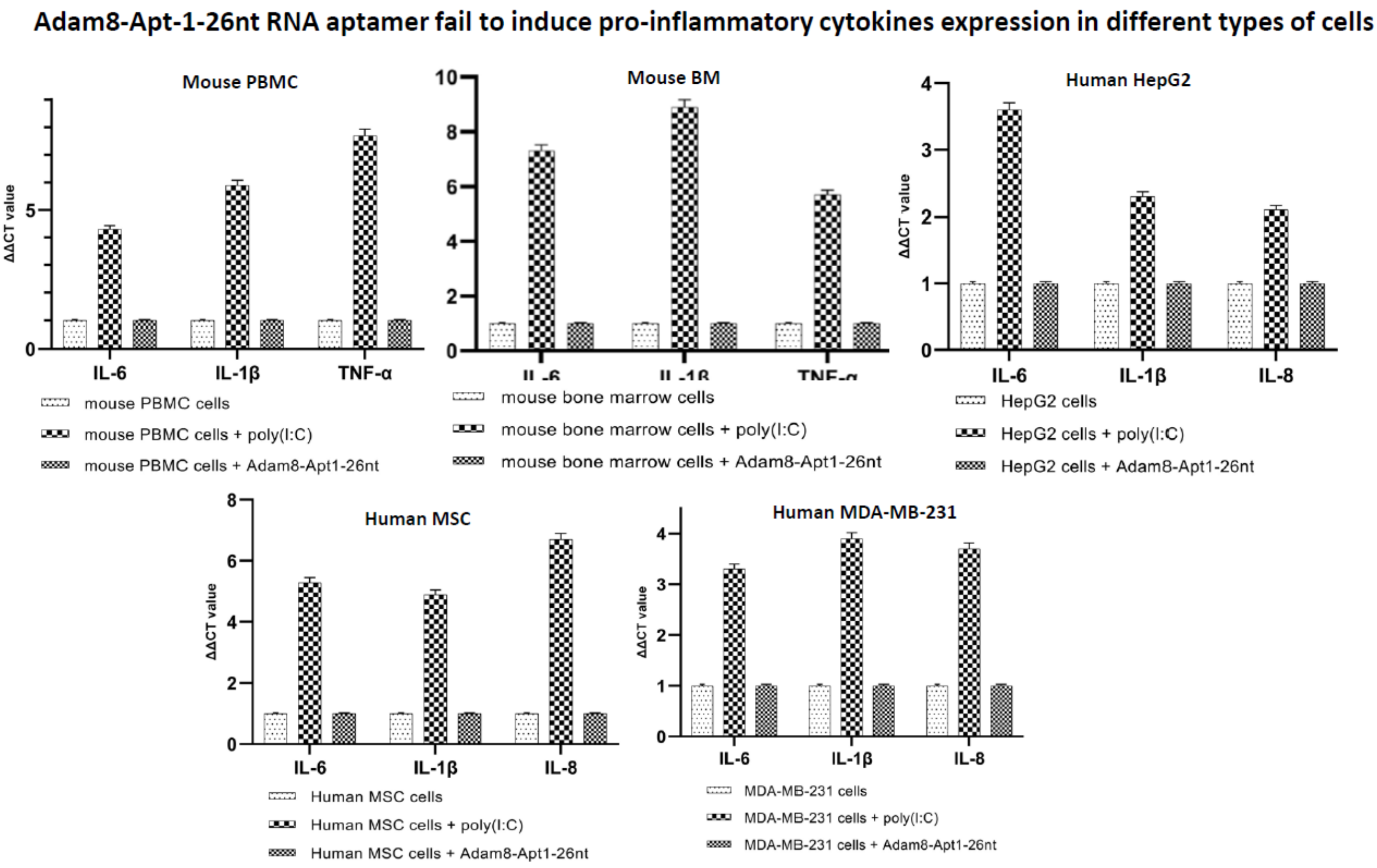

Figure S5. Adam8 Apt-1-26nt aptamer does not induce immunogenicity.

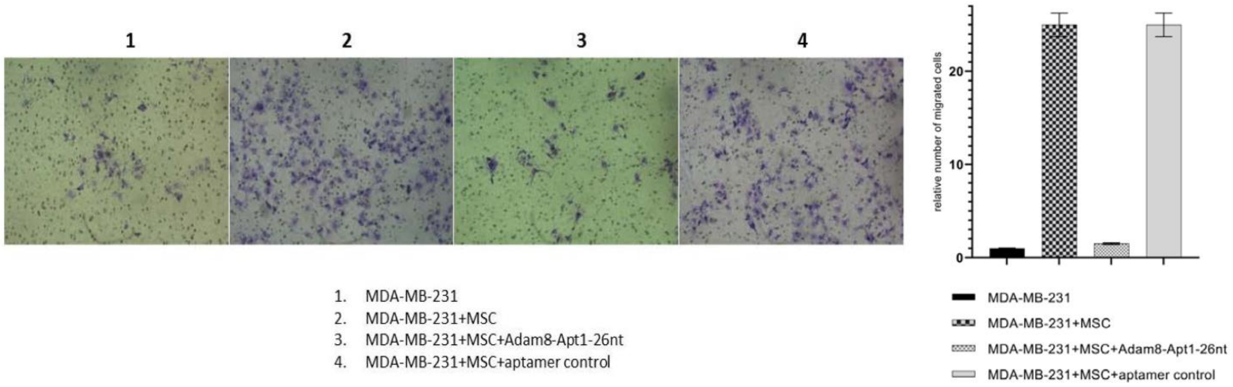

Figure S6. MDA-MB-231 cell invasion assay (16h).

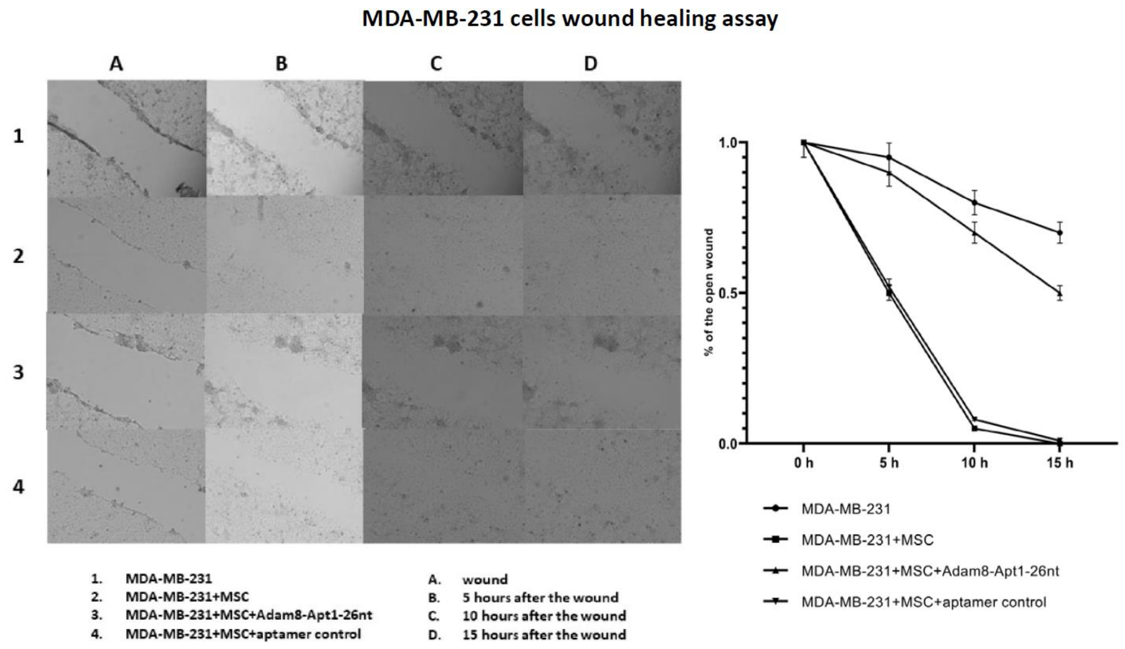

Figure S7. MDA-MB-231 cell wound-healing assay.

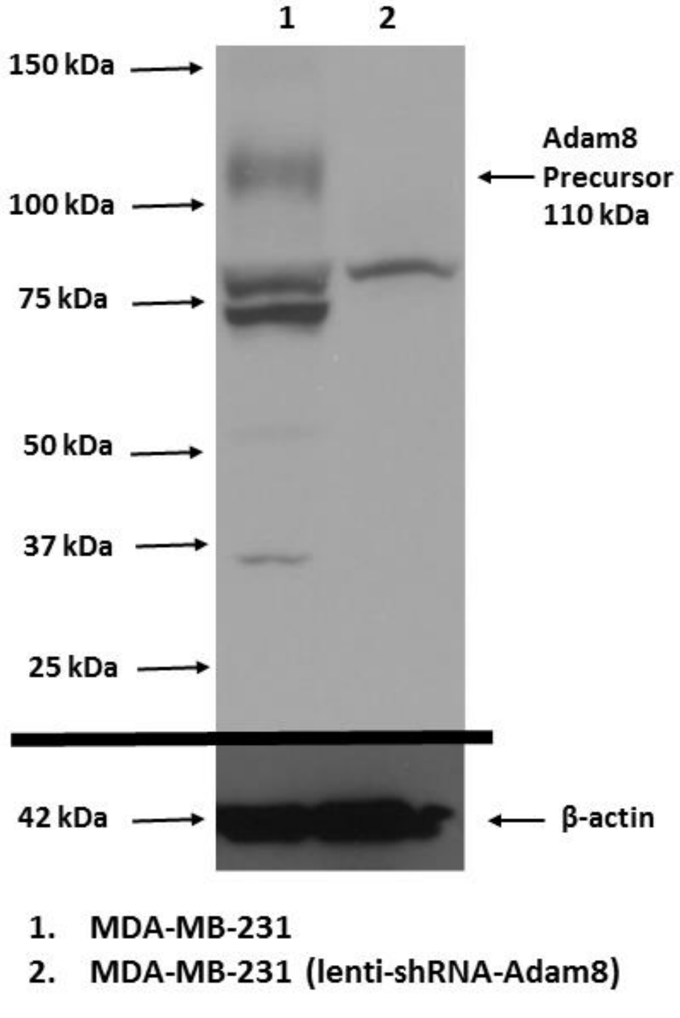

Figure S8. Adam8 Knockdown in MDA-MB-231 Cells.
